# Effect of active and passive techniques used in thromboembolic prophylaxis on venous flow velocity in the post-procedure period

**DOI:** 10.3389/fphys.2024.1323840

**Published:** 2024-03-27

**Authors:** István Zsenák, Alexandra Makai, Gabriella Kiss, Béla Faludi, Alexandra Straub, Brigitta Szilágyi, Anita Velényi, Melinda Járomi

**Affiliations:** ^1^ Doctoral School of Health Sciences, University of Pécs, Pécs, Hungary; ^2^ Institute of Physiotherapy and Sport Science, University of Pécs Faculty of Health Sciences, Pécs, Hungary; ^3^ Medical School Sports Medicine Center, University of Pécs, Pécs, Hungary; ^4^ Neurology Clinic, University of Pécs Medical School, Pécs, Hungary

**Keywords:** venous flow rate, thromboembolism prophylaxis, least squares methods, logarithmic trend line, venous flow prediction

## Abstract

**Introduction:**

Studies have shown that using mechanical thromboembolic prophylaxis methods speeds up venous flow and decreases stasis. These studies examine the post-intervention period of 1–10 min. The length of the effect of procedures to raise venous flow velocity cannot be determined by clinical trials. To apply mathematical techniques to estimate how long mechanical thromboembolism prophylaxis procedures will increase venous flow rate.

**Methods:**

In the survey, we examined 25 persons (poststroke patients), with an average age of 57.2 ± 6.3 years. Regarding the proportion of genders, 13 (52%) participants were male and 12 (48%) female. The peak venous blood flow velocity was measured with a HADECO BIDOP ES-100V II type Doppler ultrasound device, using an 8 MHz head, in the femoral vein, at the level of the hip joint. We estimated the change of the venous blood flow velocity from the available sampled data using the method of least squares. For the calculations, we used Microsoft Excel, version Mac Excel 2019.

**Results:**

The decrease in peak venous flow velocity can be approximated by a logarithm function. Mathematical calculations show that after active thromboembolic prophylaxis interventions, resting venous flow velocity is restored at 26.8 min on the intact limb and 85.1 min on the hemiparetic side. Resting flow velocity is restored in 131.9 min after passive mobilization of the hemiparetic side and in 137.7 min after the consensual effect.

**Discussion:**

An elementary mathematical function can be used to estimate the time to recovery of peak venous flow velocity to resting state from measurements taken 15 min after the intervention. Active and passive mechanical thromboembolic prophylaxis after the intervention has a longer-term effect on venous flow velocity.

## 1 Introduction

Thromboembolism prophylaxis is necessary during any disease, injury, or operation involving immobilization. Based on Virchow’s triad, the development of thrombosis is influenced by three factors: hypercoagulation, hemodynamic changes and endothelial damage. With the methods of mechanical thromboembolism prophylaxis, the hemodynamic changes can be favorably influenced by increasing the venous flow velocity, and the reduction of stasis can be achieved (Bagot and Arya, 2008).

Thromboembolism prophylaxis uses pharmacological and mechanical methods in the therapeutic treatment of patients. Mechanical methods can be divided into active and passive methods. The venous exercise therapy we perform, as well as early mobilization, is one of the active mechanical methods. As a result of movements, not only the exercised limb, but also the venous flow velocity of the passive limb increases through the consensual effect. Passive mechanical thromboembolism prophylaxis includes massage (Swedish massage, deep strokes), continuous compression (compression socks), intermittent pneumatic compression treatment, electrostimulation of calf muscle, and positioning (position during rest).

In all clinical settings, thromboembolism prevention is necessary. Combining mechanical and pharmacological thromboembolic prevention yields positive results. By accelerating venous flow and decreasing venous stasis, mechanical thromboembolism prophylaxis prevents thromboembolism (Roderick, 2005; Edwards et al., 2008; Rothberg et al., 2010). The impact of mechanical thromboembolism prevention on venous flow velocity has been examined in several research (Kwon et al., 2003; Nelson et al., 2008; Sekk et al., 2015; Kiss et al., 2019). They have shown that massage stimulates the muscles and improves their contractions, promoting venous circulation. Pressure and rubbing stimulate the blood vessel walls, increasing vessel elasticity and enhancing flow velocity. The mechanical effect of massage increases the activity of the muscle pump, providing further assistance in enhancing venous flow. It has also been demonstrated that electrical impulses applied through electrotherapy stimulate venous circulation. The procedure helps reduce muscle stiffness and improve muscle tone, promoting venous circulation. Regarding passive mobilization, it has been shown that joint movement during this procedure stimulates blood circulation, especially in the affected areas. Joint movement increases the flow of fluid between tissues, increasing venous flow velocity. In the case of elastic compression, such as the use of compression stockings, it has been demonstrated that applying pressure to the lower extremities also increases venous flow velocity. External pressure reduces the elasticity of blood vessel walls, aiding in vessel constriction and upward movement of venous blood. These studies examine the first 10–15 min following the intervention. The venous flow rate has not yet reached resting levels during this period (Stein et al., 2010, Benk 2002; Sekk et al., 2015; Kiss et al., 2019, Myron 2008).

Knowing when the venous flow rate would return to its resting level following the intervention would be helpful from a practical standpoint. Knowing this would make it easier to estimate how frequently mechanical thromboembolic prophylaxis would be required.

The patient must lie still following the procedure to determine the impact of the intervention. Due to nursing responsibilities and patient care, this is not practical for long periods of time. From the actual venous flow rate measurement data, a mathematical procedure is required to ascertain the change in venous flow rate in the post-intervention period.

To ascertain the time period following active and passive thromboembolic prophylaxis at which the venous flow rate recovers to resting values. Also to determine if the consensual effect—measured as the hemiparetic side’s venous flow velocity at rest during active movements of the intact limb—applies to the hemiparetic side’s venous flow velocity.

In the recent research, we examined elementary mathematical functions that can describe the change in venous flow velocity. We were able to represent the closest relationship with the slowing down of the venous flow rate with the logarithmic function. For each patient, we determined the equation that describes the change in the velocity of the venous flow, and made a prediction of when it will return to the resting level.

Our hypotheses are the following:

H1: It is assumed that the venous flow rate does not reach the resting venous flow rate within 20 min after the intervention.

H2: Assume that there exists an elementary function that approximates the change in the peak venous flow velocity well.

## 2 Materials and methods

### 2.1 Material and methodology

In the survey we examined 25 persons (poststroke patients), with the average age of 57.2 ± 6.3 years. Regarding the proportion of genders, 13 (52%) participants were male and 12 (48%) female. Detailed data and comorbidities of the patients are shown in Table 1. Patients having a diagnosis of hemiparetic stroke and a lower limb muscle strength grade 3 on the hemiparetic side according to the Medical Research Council 0–5 system (i.e., who are able to actively move against gravity but not against resistance, over (almost) the full range) were eligible to participate in the survey. Deep vein thrombosis, pulmonary embolism, severe circulatory disruption, medical or neurological conditions that would preclude the use of physiotherapy, and neuropathy with vascular consequences were among the exclusion criteria. The patients were examined at two locations: the University of Pécs Faculty of Health Sciences, Institute of Physiotherapy and Sport Science, and the Rehabilitation Ward for Patients with Severe Brain Injury of the Neurosurgery Department of the Clinical Center of the University of Pécs.

**TABLE 1:** Cumulative data of the study participants.

Age	Gender (%)	Type of stroke (%)	Comorbidities (%)	Time of immobility^a^
57.2 + 6.3 years	13 male, 52	ischemic stroke 16 persons, 64	hypertension 21 persons, 84%diabetes 4 persons, 16	13.5 ± 2.9 days
	12 female, 48	hemorrhagic stroke 14 persons, 56	musculoskeletal disease that does not affect the range of motion of the lower limb joints 17 persons, 68	

^a^ The number of days since the patient became bedridden.

The examinations were performed between the 2016–2022.

The research ethics approval was granted by the University of Pécs Clinical Centre Regional and Institutional Research Ethics Committee, its record number is 6129/2016.

### 2.2 Testing method

Using an 8 MHz head and the “PEAK VELOCITY” mode on a Hadeco BIDOP ES-100V II type Doppler ultrasound equipment, the peak venous blood flow velocity was determined. As in several previous studies, the venous blood flow velocity was measured at the level of the hip joint in the femoral vein (Kwon et al., 2003; Toya et al., 2016; Kiss et al., 2019; Tsuda et al., 2020). Participants wore only underwear on their lower bodies and relaxed, comfortable attire throughout the assessment. The examining room has a temperature range of 22°C–24 °C.

We examined the resting venous flow velocity in the intact leg (non-paretic) and the peak venous flow velocity after active exercise therapy. In the hemiparetic leg, venous flow velocity was measured at rest, after passive mobilization, and after active venous exercise therapy of the intact limb (consensual effect).

The following four sampling modes were therefore defined in the subsequent calculations:

**S1**: Resting venous flow velocity in the intact and hemiparetic leg.

**S2**: Effect of active mechanical thromboprophylaxis (active exercise therapy) in the intact and hemiparetic leg.

**S3**: Effect of passive mechanical thromboprophylaxis in the hemiparetic leg.

**S4**: Consensual effect in the hemiparetic leg.

### 2.3 Resting venous flow rate

Participants were positioned with a 4-cm pillow beneath their heads while laying on their backs in a horizontal position on the bed. In each case, 20 min of bedrest were taken prior to the measurements in the resting condition.

#### 2.3.1 Active mechanical thromboembolism prophylaxis of venous thromboembolism

After 20 min of rest, the participants performed vein therapy exercises according to the protocol under the guidance of a physiotherapist.

Venous exercise therapy: The structure of the exercise program is the following: breathing exercises, isometric exercises of the lower limbs, isotonic exercises of the lower limbs, ankle movements, muscle pumps, pelvis lifts, and breathing exercises in lying position. The repetition number of breathing exercises and isotonic exercises is 16. The repetition number of the isometric exercises is 16. Post-exercise peak venous flow velocity testing at 1, 2, 3, 4, 5, 6, 7, 8, 9, 10, and 15 min. The change in venous flow velocity was then determined mathematically.

Based on professional guidelines, the elements of venous exercises therapy are pump function and breathing exercises. By straining the calf, the muscle pump function is utilized, thereby increasing the venous flow velocity, and breathing exercises use the suction effect of the chest to help the circulation of the venous system towards the heart. Based on systematic literature reviews, in order to improve the dynamics of the lower limb, three to four muscle contractions per second are the most effective, but the tempo and duration of the exercises primarily depends on the patient’s condition (Katona and Siegler, 2004; Olah, 2012; Volpe et al., 2020; Li et al., 2022; Wang et al., 2023).

The literature recommends performing breathing exercises before and after venous exercises. Breathing exercises can be connected with upper limb and trunk movements (Olah, 2012).

The measurements were performed by one physiotherapist trained by a doctor.

#### 2.3.2 Passive mechanical thromboembolism prophylaxis-passive mobilization

After 20 min of rest, a physiotherapist performed fast-paced passive mobilization of the lower limb according to the protocol (Olah, 2012). Flexion-extension of the fingers, plantar and dorsiflexion of the ankle joint, circumduction of the ankle joint, and flexion-extension of the knee and hip joint were performed with 16 repetitions.

After the intervention, peak venous flow velocity testing was performed at 1, 2, 3, 4, 5, 6, 7, 8, 9, 10 and 15 min. The change in venous flow velocity was then determined mathematically.

#### 2.3.3 Consensual effect

After 20 min at rest, active vein therapy exercises of the intact limb are performed according to protocol under the guidance of a physiotherapist. After the procedure, the peak venous flow velocity is measured in the hemiparetic limb at 1, 2, 3, 4, 5, 6, 7, 8, 9, 10 and 15 min. The change in venous flow velocity was then determined mathematically.

### 2.4 Mathematical method

Based on the first 10 measurements, we wanted to estimate the result of the 15th measurement, demonstrating the correctness of the estimation algorithm. To understand the algorithm, we clarify and specify the meaning of the following physical quantities and mathematical tools for our task:

Velocity: the number of centimeters travelled in 1 s while blood is flowing.

Acceleration: the change in speed, i.e., its first derivative. In the analytical approach, the acceleration calculated in the *n*th iteration is the difference between the velocity values measured in the n+1st and the *n*th iteration.

Jerk: the change in acceleration, i.e., the second derivative of velocity. In the analytical approach, the jerk calculated in the *n*th iteration is the difference between the acceleration calculated in the n+1st iteration and the acceleration calculated in the *n*th iteration (Laczko et al., 2000).

Correlation coe(male)cient (r): coe(male)cient quantifying the closeness of the correlation. Its value varies between −1 and 1. The closer it is to 1, the closer the relationship between two variables. In scientific research, a value above 0.75 is considered a significant correlation (Ratner, 2009).

Determination coe(male)cient (*r* ^2^): which shows the percentage of the variance of variable x that is explained by the variance of variable y. The correlation coe(male)cient is obtained by taking *r* ^2^ as a square root.

Trendline: least squares prediction of the function f in Excel. There are several variants, such as linear, logarithmic, power, exponential, and polynomial (Bruce et al., 2010).

In our study, to estimate the decrease in venous flow velocity, we needed to assess what the decrease formally resembles. This allowed us to determine the type of trend line. What was clear was that the velocity decline resembles a linear function curve for a while, and then the slope breaks somewhere and the negative acceleration decreases. To define the function curve more precisely, we analyzed the change in acceleration. It is important to note that acceleration is monotonically decreasing, so in common parlance it is defined as deceleration. To determine the way in which acceleration decreases, its variation, the jerk, was investigated. From the twitch, we calculated the moving average of the difference between the last calculated twitch and the next assumed twitch, and used this to calculate the acceleration and then the velocity from the acceleration. This was done iteratively for each missing measurement point using the following solution:

$$a_{n}=v_{n+1}-\;v_{n}$$ (1)

The accelerations can be used to calculate the jerk, which is given by the formula:

$$j_{n}=a_{n+1}-\;a_{n}$$ (2)

Denote the difference between any two calculated twitches $d_{n}$ -with. In this case, our algorithm estimates the following $j_{n+1}$ :

$$j_{n+1}=j_{n}-\frac{\sum_{x=1}^{n}d_{n}}{n}$$ (3)

**FIGURE 1:**
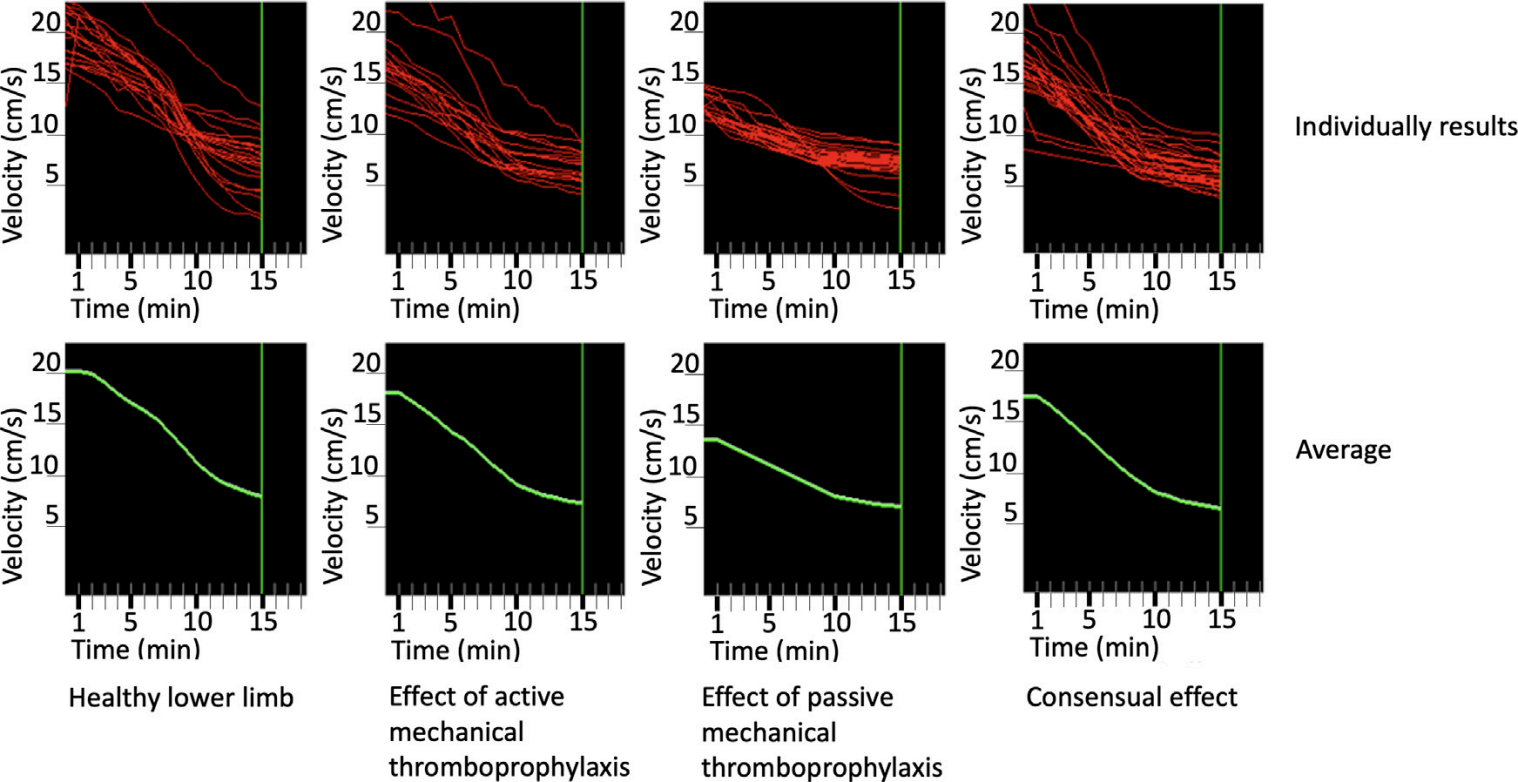
Prediction of speeds in the four different sampling modes per person and on average.

$j_{n+1}$ of the estimated value of $a_{n+1}$ -can be estimated by simply using $a_{n+1}=a_{n}-\;j_{n+1}$ formula. From there, the $v_{n+1}$ as for the following acceleration, the $v_{n+1}=v_{n}-\;a_{n+1}$ can be estimated by the formula. Our algorithm is applied to all $v_{n+1}$ value is estimated, the calculation is repeated, since the $j_{n+1}$ the elements of the sum formula used to calculate it have changed. The prediction of speeds in the four different sampling modes are shown on Figure 1.

From the calculations, we found that the velocity variations show a logarithmic pattern, so the trend line was generated using the least squares method with a logarithmic formula.

After analyzing the shape of the function, the previous algorithm was not used to fill in the missing values of sample points 11, 12, 13 and 14, because although it allowed the selection of the appropriate elementary function for the calculations, it also allowed the 15th sample point to be an estimate. For this reason, in further calculations, we took the 15th value as fixed, since we actually knew that value, and estimated the missing values from the 10th sampling point using the formula below:

$$v_{n}=\frac{v_{n-1}+v_{15}}{2}$$ (4)

**FIGURE 2:**
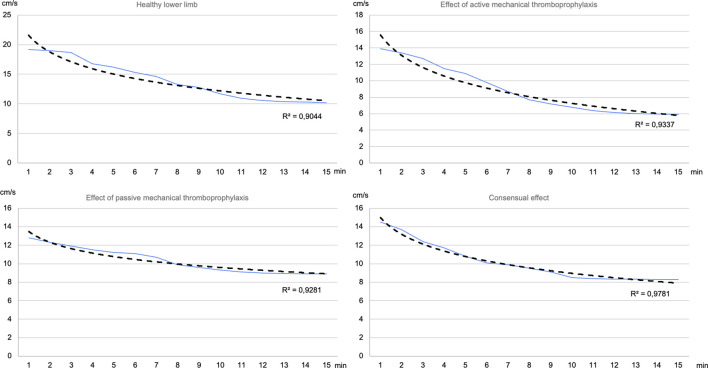
Estimation of the venous flow velocity of some randomly selected patients based on the data measured on the limb at the time of the measurement (blue line: real data and estimation, black line: logarithmic trend line calculated by Excel).

The resulting curves typically took the form illustrated in Figure 2.

The average correlation coe(male)cient between the measured data and the logarithmic trendline was 0.92 for the healthy lower limb and active mechanical thromboprophylaxis, and 0.96 for passive mechanical thromboprophylaxis and the consensual effect. It can be seen that in all cases there is a strong relationship between the variables. In addition to the averages, it should be noted that none of the samples tested had a correlation coe(male)cient below 0.75 for any of the sampling methods.

Our method confirmed our hypothesis in H2 that there exists an elementary function that represents the change in peak venous flow velocities with su(male)cient accuracy, i.e., in a closely related way by the correlation coe(male)cient.

Based on the G*Power 3.1 software *post hoc* analysis the sample size was determined as adequate (Kang, 2021). The effect sizes of the calculated estimations were summarized with *r* correlaction coefficients presented in the manuscript.

Using logarithmic trend lines, we calculated when the peak venous flow velocity would return to the resting value. In Eq. (5), the general form of the log trend line equation is illustrated:

$$y=a∙\ln\left(x\right)+k$$ (5)

where y is the value of the peak venous flow velocity in minute *x*, *a* and *k* are the technical variables needed to produce the function, which are known in each case. If we substitute the value of the resting state for y, we can express x to obtain the number of minutes at which the peak venous flow velocity equals the resting state value. This calculation was performed in all sampling modes for all patients and averages were calculated.

## 3 Results

### 3.1 Healthy limb results

After a brain injury, the patient develops hemiplegia, paralysis of one side of the body (arm and leg on the same side). The paralyzed side is called the affected side, the hemiparetic side. The non-paralyzed side is called the intact side, this we call as healthy limb. The venous flow velocity recovered to resting after 26.8 min after venous exercise therapy in the healthy limb.

### 3.2 Active mechanical thromboembolism prophylaxis results

In the hemiparetic limb, the resting flow velocity was restored after 85.3 min following active exercise.

### 3.3 Passive mechanical thromboembolism prophylaxis results

In the hemiparetic limb, resting venous flow velocity was restored after 131.9 min following passive thromboembolism.

### 3.4 Consensual effect results

After active venous exercise therapy on the intact leg, the resting venous flow rate was restored after 137.7 min in the hemiparetic limb.

The reliability of the prediction is ensured by the high correlation coefficient between the logarithm function and the sample, but in some cases the slope of the logarithm function may be so low that the convergent state characteristic of such a function occurs before the steady state is reached. In such a situation, the velocity decreases by such a small value in a single prediction iteration (in our case in minutes) that we obtain a function that is distorted and elongated compared to reality, so that in all cases our estimate is a limit on the maximum time when the peak venous flow velocity reaches the resting state. In reality, a shorter time is conceivable, since there is no mathematical procedure to determine in which cases it takes a long time to return to rest due to the shape of the function and in which cases it takes a long time to return to rest, reflecting reality. Since we are therefore not able to filter out cases where the estimate is not accurate due to the general properties of the logarithmic function, it is always advisable to consider the calculated values as upper bounds.

During every of our measurements, the threshold value did prove to be upper than 20 min, thus confirming the H1 hypothesis.

## 4 Discussion

After the treatments used during mechanical thromboembolism prophylaxis, we examined the change in venous flow velocity in poststroke patients, and then tried to estimate the decrease in venous flow velocity and the time to reach the resting level using a mathematical method.

Resting flow velocity was examined in both healthy individuals and those with medical conditions. In healthy young adults, resting venous flow velocity was measured at 10–12 cm/s (Lyons et al., 2002; Griffin et al., 2010, Oh-Yun Kwon 2003). In elderly patients awaiting knee replacement surgery, the resting venous flow velocity was measured at 9.5 cm/s (Zhuang et al., 2021). Post-stroke patients experienced values of 2.7 cm/s in the paralyzed limb and 7 cm/s in the intact limb (Kiss et al., 2019). In our survey, we measured resting venous flow velocity at 3.2 cm/s in the paralyzed limb and 7.3 cm/s in the intact limb of stroke patients. The values we obtained for resting venous flow velocity differ from those measured in healthy adults, which can be explained by anatomical and pathomechanical factors. The surveyed patients, being bedridden and with reduced muscle pump function due to muscle paralysis, showed data to those measured in a similar patient group.

Following passive thromboembolism prophylaxis methods, venous flow velocity increased to varying degrees. Compression stockings resulted in the smallest increase in venous flow velocity, measuring 10.1–13.9 cm/s (Jamieson et al., 2007; Zhuang et al., 2021). After passive mobilization, an increase of 12.6 cm/s was observed (Kiss et al., 2019). After positioning during rest, values of 22.8–44.7 cm/s were measured (Toya et al., 2016). Neuromuscular electrical stimulation of the calf muscles led to venous flow velocity ranging from 43 to 105 cm/s (Lyons et al., 2002; Griffin et al., 2010). In our survey, rapid passive mobilization was performed on the hemiparetic lower limb of stroke patients, resulting in a peak venous flow velocity of 12.2 cm/s. Our results were similar to those obtained in a similar patient group.

After active thromboembolism prophylaxis methods, healthy young adults experienced an increase in peak venous flow velocity to 20.7–43.0 cm/s following ankle pump exercises (Toya et al., 2016, Oh-Yun Kwon 2003), which could be further increased to 69.0 cm/s with the use of an exercise band (Tsuda et al., 2020). Post-stroke patients measured a peak venous flow velocity of 18.0 cm/s following active venous exercises (Kiss et al., 2019). In our survey, stroke patients’ paralyzed lower limb measured 15.6 cm/s, and the intact lower limb measured 18.1 cm/s in peak venous flow velocity after active venous exercises. Our results differ from those measured in young adults because elderly stroke patients lack the range of ankle motion and calf muscle strength required for a significant increase in peak venous flow velocity. The values obtained in our survey are similar to those measured in stroke patients.

We examined the degree of consensual effect, where venous exercises were performed on the intact limb of post-stroke patients. After exercising the intact limb, we measured the change in venous flow velocity in the paralyzed limb. In our survey, we obtained a value of 15.8 cm/s. Kiss and colleagues investigated the consensual effect in stroke patients, measuring a value of 15.1 cm/s in our survey. Our results were similar to those examined by others in terms of resting venous flow velocity and peak venous flow velocity after interventions.

The frequency of venous exercise therapy (how many times it must be repeated during the day) is not determined by professional protocols or guidelines, because until now there has been no research in which it was possible to determine when the venous flow rate returns to the resting flow value after a mechanical thromboembolism prophylaxis. If we know when the venous flow rate returns to the resting value, we can tell how often the methods of mechanical thromboembolism prophylaxis should be applied. The previous measurement protocols gave the opportunity to provide data on the change in venous flow rate in the minute or minutes following the intervention. What we see from these studies is that venous flow velocity does not return to resting levels within 15 min after the intervention. So far, there has been no data on how many minutes the flow rate returns to the resting level. The measurement methods used so far were not suitable for monitoring the change in the venous flow rate over a period longer than 15 min. We have developed a measurement method that can be used to determine when the venous circulation returns to the resting level after the intervention. Based on this, it can be determined how often the methods of passive mechanical thromboembolism prophylaxis must be repeated. With the measurement method we have developed, it is possible to specify how often it is advisable to repeat the active mechanical thromboembolism prophylaxis methods during the day. Our results can be incorporated into the professional protocol and patient education. After learning the exercises, the patient can perform the active techniques several times a day without the presence of a physiotherapist or nurse. Based on the information received about the consensual effect, the patient can achieve increased venous circulation in the paralyzed lower limb with the non-paralyzed (actively moving) limb. Based on the survey, the duration can be specified, how often the exercise should be done during the day. Thus, after education, the patient can achieve increased circulation in the paralyzed limb, several times a day without the help of a physiotherapist or nurse. This can be more effective in preventing thromboembolic complications during immobilization (long-term bed rest).

## Data Availability

The original contributions presented in the study are included in the article/Supplementary material, further inquiries can be directed to the corresponding author.
